# Inequity on energy and nutrient consumption among urban residents of Ethiopia may impact the effectiveness of large-scale food fortification

**DOI:** 10.1017/S0007114526107296

**Published:** 2026-04-29

**Authors:** Semira Mitiku Saje, Louise Ander, Elaine L. Ferguson, Dawd Gashu

**Affiliations:** 1 Center for Food Science and Nutrition, Addis Ababa University, Addis Ababa, Ethiopia; 2 School of Biosciences, University of Nottingham, Sutton Bonington Campus, Loughborough, Leicestershire, UK; 3 Inorganic Geochemistry, Centre for Environmental Geochemistry, British Geological Survey, Nottingham, UK; 4 Department of Population Health, London School of Hygiene & Tropical Medicine, London, UK; 5 Agriculture, Nutrition and Health Academy Science-Policy Fellowship, Friedman School of Nutrition Science and Policy at Tufts University, Boston, MA, USA

**Keywords:** Food fortification, Gender inequity, Nutrient intakes, Micronutrients, Dietary requirements, Nutrition intervention, Ethiopia

## Abstract

Intra-household energy and nutrient inequity occur when food is distributed differentially, leaving some members without adequate nutrition. If unaddressed, this can reduce the effectiveness of food based nutrition interventions. This study assessed energy and micronutrient intake inequities among urban Ethiopian households before and after wheat flour and edible oil fortification. Using 24-h dietary recall data from the 2013 Ethiopian Household Food Consumption Survey, we analysed 375 households with adult men and women. The Ethiopian mandatory fortification standard for wheat flour and edible oil was considered in the simulation. Usual intake estimates and fortification modelling were performed using the Simulating Intake of Micronutrients for Policy Learning and Engagement macro 1-day method. Inequity ratios were calculated by comparing energy and nutrient intakes with dietary requirements for each group. Prefortification, the median nutrient consumption was generally higher among men, except for vitamin A. Fortification of wheat flour with Zn and vitamin B_1_, along with vitamin A-fortified vegetable oil, led to a 26–74 percentage point reduction in nutrient inadequacy among all participants. Men could benefit more from Zn and vitamin A fortification, whereas women could benefit more from vitamin B_1_ fortification. Inequity estimates before and after fortification ranged from 1 to 1·5, favouring men. Although intra-household food allocation was not directly assessed, observed differences in nutrient intake between men and women suggest gender-related disparities that should be considered in nutrition interventions.

Intra-household inequity in food access occurs when different members of a household have unequal access to and control over food resources, leading to disparities in nutritional outcomes^(1)^. Understanding intra-household food and nutrient distribution is crucial for policymakers in identifying vulnerable groups, using household food consumption data and planning effective nutritional interventions such as food fortification^(2)^. Food fortification is an important strategy for improving nutrient intakes, particularly when individuals at risk for nutrient deficiencies consume fortified foods in the amounts predicted when setting fortification levels. However, fortification must also ensure safety by avoiding excessive nutrient intakes beyond safe levels among individuals at risk of overconsumption^(3)^.

Gender inequity is a prominent dimension of intra-household food distribution, influenced by cultural norms, food preference, gender roles and power dynamics^(4–6)^. Cultural beliefs, mainly in resource poor countries, often prioritise males in intra-household food distribution, disadvantaging women and girls^(5)^. Gender roles and decision-making power further exacerbate these disparities, with men typically controlling household resources, affecting women’s ability to access nutrient-rich foods^(7)^. Economic disparities and limited nutritional awareness also contribute to energy and nutrient inequitable food allocation within households, impacting the health of vulnerable groups, particularly women and children^(8)^.

Evidence on intra-household gender-related energy and nutrient inequities is mixed. While some studies suggest that men receive culturally supported preferential treatment in food distribution, others indicate that women may, at times, be more advantaged^(9–11)^ For example, a study in Ethiopia by Coates et al.^(7)^ found that women had equity in protein intake and were favoured in energy distribution compared with men, while Fe intake better meets the needs of men. Similarly, Villa et al.^(12)^ reported that daughters had higher dietary diversity than other family members, whereas sons were at a disadvantage. However, a systematic review of twenty-eight studies by Berti^(13)^ found no consistent pattern regarding which household members benefit most from energy equity.

Ethiopia has mandated the large-scale fortification of wheat flour and edible oil during 2022^(14)^. Differential food allocation within households may undermine its effectiveness – either by failing to reach the intended beneficiaries in adequate amounts or by causing nutrient excess among others when a fortified food vehicle is consumed in larger quantities than expected^(13)^. Therefore, this study aimed to assess differences in Zn, Ca, vitamin A and vitamin B_1_ consumption relative to average requirements between adult men and women in urban Ethiopian households. It also sought to simulate the impact of wheat flour and edible oil fortification on nutrient adequacy and gender equity in these populations.

## Methods

### Data source

We extracted data from the 2013 Ethiopian National Food Consumption Survey for households (*n* 375) with both an adult man and women (except pregnant women). The survey was conducted in all nine regions and two city administrations. The survey included male participants only from urban areas to assist in setting the safe upper level of fortification because they are likely to have access to and consume the highest amount of any selected fortifiable food^(15)^; therefore, the present study analysed dietary intake data from urban households only, which are not nationally representative.

Details about the Ethiopian National Food Consumption Survey methodology were published elsewhere^(15)^ and are briefly summarised here. Dietary data were collected using the multiple-pass 24-h dietary recall method^(16)^. Each participant provided a single-day recall of their dietary intake, and portion size estimate was done using digital food scale, household utensils, food substitutes (playdough, flour, lentils and water) and pictures of most commonly consumed foods^(15)^. The energy and nutrient content of consumed foods were estimated by matching to the Ethiopian food composition table volume III and IV and other published studies^(17–22)^.

To estimate usual energy and nutrient intake, we employed the Simulating Intake of Micronutrients for Policy Learning and Engagement macro using the 1-day method. This statistical method uses single-day dietary data and an external within-person to between-person variance ratio to estimate population distributions of usual intake, thereby approximating long-term usual intake distributions at the population level^(23)^. The Simulating Intake of Micronutrients for Policy Learning and Engagement macro is particularly suitable for settings where repeated dietary recalls are not available, as it enables robust estimation of nutrient inadequacy and excess using limited data^(24,25)^.

The in-person variance for the present analysis was obtained from Uganda for Zn (0·61), Ca (0·64) and vitamin A (0·4) and Burkina Faso for energy (0·82) and vitamin B_1_ (0·84)^(24)^. The percent energy contribution from fortifiable wheat flour (industrially processed wheat flour and foods made from such flour) and vegetable oil (imported or locally produced by facilities with refineries) was calculated by multiplying the amount consumed by their respective nutrient contents and dividing the result by the total intake.

### Fortification simulation

We simulated the impact of fortifying wheat flour with Zn and vitamin B_1_ (thiamine) and edible oil with vitamin A, for both genders. The addition rates at the factory level and expected activity loss were based on fortification specification document from the Ethiopian Standards Agency. After adjusting for loss the addition rate was 0·044 mg for Zn, 0·008 mg for vitamin B_1_ per gram of wheat flour and 28 μg RAE for vitamin A per gram of oil^(26,27)^. Ca is not included in the Ethiopian food fortification standard and was therefore excluded from the simulation; however, it is presented in this report because of its public health importance. In contrast, although folate is included in the wheat flour fortification standard, insufficient data were available to model its fortification impact.

To estimate the risk of energy and nutrient inadequacy or excess among the study participants; first, usual energy and nutrient intakes were estimated using the Simulating Intake of Micronutrients for Policy Learning and Engagement macro with the 1-day method^(28)^. This approach adjusts single-day dietary intake data for within-person variability using externally derived variance components to approximate usual intake distributions. Second, daily energy requirements were calculated assuming physical activity level of 1·6, as recommended by FAO in the absence of population-specific data^(29)^ FAO, and reference adult body weights of 60 kg for men and 55 kg for women^(30)^. The present study assumed consumption of an unrefined diet and, consequently, low mineral bioavailability^(31)^. Inadequacy was assessed using the estimated average requirement (EAR), while excess intake was assessed using the upper tolerable intake level where available. Estimates of inadequacy and excess were calculated separately for men, non-pregnant non-lactating women, and lactating women according to their respective physiological requirement profiles.

The inequity of consumption was assessed by calculating the male-to-female inequity ratio for energy and nutrient intake. This process included the following steps: First, the median intake and EAR were summed for pregnant and non-pregnant women, each with distinct EAR. The same procedure was repeated for males. Next, the total nutrient intake for each gender was divided by their respective total EAR to determine the intake:EAR ratio for both men and women. Finally, the male-to-female inequity ratio was obtained by dividing intake:EAR ratio of male and female^(7)^. A ratio below 1 (< 1) signifies inequity in favour of females, while a ratio above 1 (> 1) indicates inequity in favour of males.

## Results

Table 1 presents the characteristics of the study households. We studied 375 households, each with one adult male and one women of reproductive age. Half of these households were located in Addis Ababa, Dire Dawa and the Harar regions, which unlike the other regions are predominantly urban areas. More than 90 % of the households in this study fall into the highest two quintiles of socioeconomic status according to the national classification. Approximately equal proportions of the women were lactating and non-lactating non-pregnant women and a relatively low percent were pregnant, whilst 89 % of households were male headed.

### Fortifiable foods, energy and nutrient intake

Median consumption of fortifiable wheat flour was 235 g/d for men and 176 g/d for non-pregnant women. Fortifable edible oil consumption was 20·7 g/d for males and 18·5 g/d for non-pregnant women. Fortifiable wheat flour could contribute 27·2 % and 26·8 % of total energy intake for men and women, respectively, while fortifiable edible oil could contribute 11·7 % for men and 12·3% for women.

Fortifiable wheat flour and edible oil made meaningful contributions to daily micronutrient requirements. Fortified wheat flour could contribute approximately 63·0 % of the recommended Zn intake for men and 47·4–65·7 % for women and about 150·0% and 100·0–133·3 % of the recommended vitamin B 1 intake for men and women, respectively. Meanwhile, fortifiable edible oil could contribute approximately 89·7 % of the recommended vitamin A intake for men and 47·9–99·7 % for women. Despite mean vitamin B 1 intakes exceeding reference values after fortification, approximately 12·4 % and 12·8 % inadequacy persisted for men and non-pregnant women, respectively, reflecting inter-individual variability in fortified wheat flour consumption.

Prefortification median intakes of energy and most nutrients were generally higher among men than among women, with the exception of vitamin A. For vitamin A, non-pregnant and non-lactating women had higher median intakes, while intakes were comparable between men and lactating women. Excess consumption was less than 0·1 % for all nutrients in both groups (Table 2).

The simulation was conducted for vitamin A (in vegetable oil) and for Zn and vitamin B_1_ in wheat flour, as these are the nutrients included in the Ethiopian fortification standard. The simulation of wheat flour and edible oil fortification demonstrated a 26–74 percentage point reduction in nutrient inadequacy, with males showing the highest reduction in Zn and vitamin A deficiency, while women benefited more from vitamin B_1_ fortification (Table 2). Excess consumption rates were 1·5 % and 2 % for vitamin A and 7·6 % and 19·7 % for Zn among women and men, respectively. There is no scientifically established upper intake level defined for vitamin B_1_.

### Inequity ratio

Table 3 shows gender disparities in nutrient intake before and after fortification, as reflected by the men-to-women intake ratios relative to the EAR. Men generally had higher median intakes of Zn and vitamin B_1_ than women across all household types, with ratios ranging from 1·0 to 1·5 both before and after fortification. In contrast, women had relatively higher vitamin A intake before fortification, particularly in households with lactating women, whereas men had higher intakes after fortification. The simulated fortification had little effect on reducing gender inequity for Zn and vitamin B_1_ but substantially altered the pattern for vitamin A, especially in households with lactating women.

## Discussion

Gender inequity in food and nutrient consumption can undermine the effectiveness of food fortification programs by limiting their reach among vulnerable groups while increasing the risk of excess nutrient intake among already advantaged individuals^(13)^. This study estimates gender disparities in the intake of energy, Zn, Ca, vitamin A and vitamin B_1_ among a non-nationally representative survey of urban adult men and non-pregnant women living in the same households in Ethiopia. Prefortification, the prevalence of inadequacy exceeded 76 % for most nutrients in both groups, except for vitamin B_1_, which was 17 % for men and 41 % for women. Simulations of wheat flour fortification with Zn and vitamin B_1_, along with vitamin A fortification in edible oil, suggested potential reductions in nutrient inadequacy by 27–69 percentage points. Despite these improvements, inequity ratios indicated that men remained more advantaged.

The median energy consumption for men in our study was estimated at 1774 kcal/d, closely aligning with the national food consumption survey’s estimate of 1762 kcal/d. In contrast, a discrepancy was noted for women, with our study estimating 1441·6 kcal/d compared with 1726 kcal/d. Zn intake for non-pregnant urban women was estimated at 7·1 mg/d in our study, the national estimate was 7·4 mg/d for urban women^(13)^. For men, median Zn intake was 9·5 mg/d, while regional estimates from Addis Ababa and Somali indicated 8·2 mg/d^(32)^. Ca intake also showed a difference, with non-pregnant women in our study having a median of 352·2 mg/d, compared with 300 mg/d from the national survey^(15,33)^. For vitamin A, the median consumption was estimated at 54 μg/d for men and 57 μg/d for non-pregnant women in our study, compared with 39 μg/d in the national survey^(15)^. Though all three studies came from the same data source, differences can be attributed to the distinct focus groups of each study, as the national survey covered a broader population, while our study specifically included urban, households because these are the only populations with data for both men and women in the same household.

The fortification simulations suggest that wheat flour and edible oil fortification could substantially reduce the prevalence of micronutrient inadequacy among both men and non-pregnant women. Before fortification, Zn inadequacy affected 76·5 % of men and 89·1 % of non-pregnant women, comparable to previous national estimates of 83 % among men and 85 % among women of reproductive age^(32)^. Following simulated fortification, Zn inadequacy could decline to 27·3 % among men and 42·2 % among non-pregnant women, indicating a marked potential improvement in Zn intake, although gender disparities would likely persist. Similarly, vitamin A inadequacy was very high at prefortification (96·0 % in men and 97·3 % in non-pregnant women), consistent with reports among adolescent girls^(16)^, but substantially higher than the low deficiency prevalence reported among women of reproductive age in the national micronutrient survey^(15)^. After simulated fortification with vitamin A-fortified oil, inadequacy could decrease to 50·3 % in men and 62·5 % in women, demonstrating a considerable potential contribution of fortification to improving vitamin A intake, albeit with remaining gaps.

For vitamin B_1_, baseline inadequacy was 16·9 % among men and 41·1 % among women, falling within the wide range reported in previous studies^(34)^. The simulation indicates that fortification could reduce vitamin B_1_ inadequacy to similarly low levels among men (12·4 %) and women (12·8 %), suggesting a more equitable potential impact for this nutrient. This variability is influenced by differences in dietary intake during fasting and non-fasting seasons, as well as the lactating mother’s fasting status, especially when thiamine-rich animal source foods are limited^(34)^. In addition, variability in vitamin B_1_ status may reflect food processing practices that reduce thiamine content, particularly in settings with limited or no food fortification, as well as differences in household food security, socio-economic status and dietary diversity. Health-related factors, including infections, gastrointestinal disorders and increased physiological demands during lactation, may further contribute to inadequate thiamine status^(35–37)^. Ca inadequacy was notably high, with 97 % for men and 100 % for non-pregnant women in our study, consistent with findings from other studies, which reported rates between 97·5 % and 100 % for women and adolescent girls^(16,34)^.

The fortification simulations suggest that wheat flour and edible oil fortification could reduce gender-related inequities for some nutrients. Specifically, the Zn intake inequality ratio could decrease from 1·23 before fortification to 1·17 after fortification, and the vitamin B_1_ ratio from 1·30 to 1·20, indicating a relative improvement in intake among non-pregnant women. In contrast, the vitamin A intake ratio could increase from 0·99 to 1·17 following fortification, indicating a shift from near parity pre-fortification to higher intake among men. Overall, these findings suggest that fortification could contribute to modest improvements in equity for Zn and vitamin B_1_, while potentially increasing disparities for vitamin A, underscoring the importance of incorporating equity considerations into fortification program design. Similarly, a study reported a 0·76 women:men ratio for iron consumption, indicating less favourable intake for women^(7)^. Vitamin A intake estimates varied between households with nonpregnant non-lactating women and those with lactating women. In the former, women appeared to be favoured, whereas in the latter, men were favoured. This disparity is primarily due to the substantially higher EAR for lactating women (1020 μg/d RAE)^(31)^. In addition, sociocultural and household factors, including large family size, low dietary diversity, household food insecurity and limited antenatal care attendance, could lead to inadequate consumption of vitamin A-–rich foods^(38)^. Intra-household feeding hierarchies and social norms, such as women eating after men, may further restrict women’s access to vitamin A–rich foods^(39,40)^.

The strengths of the study include its contribution to understanding gender disparities in nutrient intake in Ethiopia by focusing on urban settings, thereby shedding light on the dietary challenges faced by urban population groups. Additionally, it utilises advanced modeling techniques to simulate the impacts of wheat flour and edible oil fortification, providing an analysis of the potential benefits for different gender groups. The study also has limitations that need to be acknowledged. First, the number of households included was not representative of urban households in Ethiopia. Consequently, the results of this paper are reflective only of the sampled households. Second, usual intake was estimated by borrowing within-person variation from other studies, which might affect the accuracy of our estimates. However, we used variances that had previously been applied in analyzing the national survey and conducted sensitivity analyses to minimise potential biases. Third, the energy estimates appear to be underestimated. This also affects nutrient intake estimates, potentially leading to an overestimation of inadequacy. However, the degree of energy underestimation seems consistent across both groups.

In this study, usual intake distributions were estimated using within-person variance components derived from dietary surveys conducted in Uganda and Burkina Faso. This approach is commonly applied when repeated dietary measurements are unavailable^(28)^. However, it introduces important methodological limitations. Within-person variability in dietary intake is influenced by cultural food practices, seasonality, market access, dietary diversity and socioeconomic conditions^(24,41)^. Consequently, variance components obtained from external populations may not accurately reflect the true intake variability in the Ethiopian context. Nevertheless, in the absence of nationally representative multiple-day dietary data, the use of external variance estimates represents a pragmatic solution and is consistent with established approaches in population-level dietary assessment.

To improve the accuracy of usual intake estimation in future studies, collecting repeated 24-h dietary recalls from a subsample of the population would allow direct estimation of within-person variability should be considered^(41)^. In addition, incorporating seasonal dietary surveys would help capture temporal variability in intake patterns^(42)^.

In conclusion, the prefortification data revealed gender inequity in Zn, Ca and thiamine consumption within the studied households, consistently favouring men, with the most significant disparity observed in vitamin A intake in lactating women households. Furthermore, fortification simulations showed improvement in nutrient intake and a reduction in inadequacy prevalence for men. These findings provide evidence for policymakers to integrate gender disparities when designing targeted nutrition interventions aimed at addressing nutrient intake.

## Figures and Tables

**Table 1.** Characteristics of study urban households from the Ethiopian National Food Consumption Survey, 2013

Variable		*n*	%
Sex	Men	375	50
	Women	375	50
Region	Addis Ababa	85	22·7
	Afar	9	2·4
	Amhara	33	8·8
	Benshangul Gumuz	20	5·3
	Dire Dawa	60	16
	Gambella	12	3·2
	Harari	45	12
	Oromia	27	7·2
	SNNPR	33	8·8
	Somali	24	6·4
	Tigray	27	7·2
Wealth index	Highest	263	70·1
	Upper middle	87	23·2
	Middle	13	3·5
	Lower middle	7	1·9
	Lowest	5	1·3
Women physiology	Pregnant	24	6·4
	Lactating	177	47·2
	Non pregnant and non lactating	174	46·4
Household head	Male	333	88·8

Wealth index was constructed using principal component analysis (PCA) of household asset ownership and living condition variables; resulting scores were used to rank households and classify them into five quintiles.

**Table 2.** Simulated energy and nutrient intakes among urban men and non-pregnant women before and after wheat flour and edible oil fortification in urban Ethiopia, 2013

		Before fortification						After fortification						Contribution of fortification to EAR (%)	
		
		Households with NPNLW		Households with LW		Total Households		Households with NPNLW		Households with LW		Total Households			
							
Energy & nutrients		Men	Women	Men	Women	Men	Women	Men	Women	Men	Women	Men	Women	Men	Women

Energy	Median (kcal/d)	1573·9	1387·4	1886·6	1522·9	1774·1	1441·6	–	–	–	–	–	–	–	–

Zn	Median (mg/d)	8·0	6·4	10·1	7·6	9·5	7·1	14·8	‘13·0	19·2	14·8	17·5	13·6	63·0	47·4–65·7
	
	Inadequacy %	79·9	78·6	72·4	94·1	76·5	89·1	39·3	35·45	23·5	44·7	27·3	42·2	–	–

Ca*	Median (mg/d)	366·1	335·9	449·9	374	423·8	352·2	–	–	–	–	–	–	–	–
	
	Inadequacy %	94·6	97·9	97·5	99·8	96·9	99·7	–	–	–	–	–	–	–	–

V- A	Median (μg/d)	31·0	41·5	60·2	61·1	54·2	57·5	320·6	398·1	681·9	655·1	565·3	545·8	89·7	47·9–99·7
	
	Inadequacy %	90·4	87·4	97·2	99·5	96	97·3	63·7	55·2	44·1	68	50·3	62·5		

V-B_1_	Median (mg/d)	1·3	1·2	1·5	1·2	1·4	1·2	2·6	2·4	3·2	2·6	2·9	2·4	150	100–133·5
	
	Inadequacy %	23·4	27·2	15·4	47·8	16·9	41·1	17·3	17·4	9·7	12·2	12·4	12·8		

NPNLW, non pregnant non-lactating women; LW, lactating women; estimated average requirement – Zn (low bioavailability): 12·7 mg/d for men, 9·9–13·7 mg/d for women; Ca: 750 mg/d for men, 750–960 mg/d for women; vitamin A: 570 μg/d for men, 490–1020 μg/d for women; vitamin B_1_:1 mg/d for men, 0·9 and 1·2 mg/d for women. Allen *et al*.^(31)^.

* Ca is not in the Ethiopian Food fortification standard.

**Table 3.** Inequity ratio of energy and nutrients between men and women participants before and after fortification

Energy and nutrients	Men to women nutrient intake ratio before fortification			Men to women nutrient intake ratio after fortification		
	Households with NPNLW	Households with LW	Total households	Households with NPNLW	Households with LW	Total households

Energy	0·9	1·17	1·11	–	–	–

Zn	1·03	1·46	1·23	0·91	1·47	1·17

Ca	1·41	1·5	1·49	–	–	–

Vitamin A	0·52	1·74	0·99	0·61	2	1·17

Vitamin B_1_	1·11	1·49	1·3	1	1·5	1·28

NPNLW, non pregnant non lactating women; LW, lactating women.

Consumption inequality was assessed by calculating the male-to-female inequity ratio for energy and nutrient intake. The male-to-female inequity ratio was obtained by dividing intake-to-estimated average requirement ratio of male and female. A ratio below 1 signifies inequity in favour of females, while a ratio above 1 indicates inequity in favour of males.

## Abbreviations:

EAR: estimated average requirement
